# A New Approach of Personality and Psychiatric Disorders: A Short Version of the Affective Neuroscience Personality Scales

**DOI:** 10.1371/journal.pone.0041489

**Published:** 2012-07-26

**Authors:** Jean-Baptiste Pingault, Bruno Falissard, Sylvana Côté, Sylvie Berthoz

**Affiliations:** 1 Research Unit on Children’s Psychosocial Maladjustment, University of Montreal, Sainte–Justine Hospital, Montreal, Quebec, Canada; 2 International Laboratory for Child and Adolescent Mental Health Development, University of Montreal, Montreal, Quebec, Canada; 3 INSERM U669, University Paris-Descartes and Paris-Sud, Paris, France; 4 Department of Psychiatry for Adolescents and Young Adults, Institut Mutualiste Montsouris, Paris, France; University of Medicine & Dentistry of NJ - New Jersey Medical School, United States of America

## Abstract

**Background:**

The Affective Neuroscience Personality Scales (ANPS) is an instrument designed to assess endophenotypes related to activity in the core emotional systems that have emerged from affective neuroscience research. It operationalizes six emotional endophenotypes with empirical evidence derived from ethology, neural analyses and pharmacology: PLAYFULNESS/joy, SEEKING/interest, CARING/nurturance, ANGER/rage, FEAR/anxiety, and SADNESS/separation distress. We aimed to provide a short version of this questionnaire (ANPS-S).

**Methodology/Principal Findings:**

We used a sample of 830 young French adults which was randomly split into two subsamples. The first subsample was used to select the items for the short scales. The second subsample and an additional sample of 431 Canadian adults served to evaluate the psychometric properties of the short instrument. The ANPS-S was similar to the long version regarding intercorrelations between the scales and gender differences. The ANPS-S had satisfactory psychometric properties, including factorial structure, unidimensionality of all scales, and internal consistency. The scores from the short version were highly correlated with the scores from the long version.

**Conclusions/Significance:**

The short ANPS proves to be a promising instrument to assess endophenotypes for psychiatrically relevant science.

## Introduction

### Endophenotypes in Biological Psychiatry

Recent advances in biological psychiatry suggest that several psychiatric disorders share common processes pertaining to emotional regulatory dysfunctions, and that diagnoses informed by intermediate markers of brain dysfunction - and not on the basis of overt phenotypes or syndromic behaviors – may account for these commonalties. Accordingly, there is a growing consensus to consider that to better understand the etiology of psychiatric disorders, one strategy is to study endophenotypes, i.e. ‘*measurable components unseen by the unaided eye along the pathway between disease and distal genotype*’ [1]. In order to be useful for psychiatrically relevant science, such endophenotypes should be closely linked to brain systems and genetic underpinnings [2]–[5]. Along these lines, Panksepp and coll. proposed a new theoretical framework to identify emotional endophenotypes, based on ethology, neural analyses and pharmacology. They emphasized that basic emotions – e.g. fear – are strongly linked to specific functional sub-cortical neural systems, homologous in all mammalian brains [4], [6]–[9]. These systems are a legacy of evolution and tend to elicit behavioral responses that efficiently reflect underlying emotions (e.g. fear and flight). Although the presence of these systems across species demonstrate their adaptative value, an imbalance in such systems may cause significant psychiatric and/or personality disorders [4].

### Development of the Affective Neuroscience Personality Scales and Information to Date on the Properties of the Instrument

Davis, Panksepp, & Normansell [2] operationnalized this theoretical framework with a self-report questionnaire, the Affective Neuroscience Personality Scales (ANPS) which evaluates six emotional endophenotypes: PLAYFULNESS/joy, SEEKING/interest, CARING/nurturance, ANGER/rage, FEAR/anxiety, and SADNESS/separation distress. Panksepp and coll. conceptualized PLAYFULNESS as having fun vs. being serious, playing games with physical contact, humor, and laughter, and being generally happy and joyful; SEEKING as feeling curious, feeling like exploring, striving for solutions to problems and puzzles, positively anticipating new experiences, and a sense of being able to accomplish almost anything; CARING as nurturing, being drawn to young children and pets, feeling softhearted toward animals and people in need, feeling empathy, liking to care for the sick, feeling affection for and liking to care for others, as well as liking to be needed by others; ANGER as feeling hotheaded, being easily irritated and frustrated, experiencing frustration leading to anger, expressing anger verbally or physically, and remaining angry for long periods; FEAR as having feelings of anxiety, feeling tense, worrying, struggling with decisions, ruminating about past decisions and statements, losing sleep, and not being typically courageous; SADNESS as feeling lonely, crying frequently, thinking about loved ones and past relationships, and feeling distress when not with loved ones [2].

The elaboration of the ANPS was based not only on ethological considerations, but also on pharmacological and neural studies which established that these core emotional systems have distinct - though partly overlapping – subcortical networks [4], [7]–[9]. As such, the ANPS traits do not stem from a psychometric grouping of lexical descriptors of personality, constrasting with personality scales such as the Five Factor Model (FFM) [10]. This characteristic might provide new insights as illustrated by Davis & Panksepp [8] with the Agreebleness construct of the FFM and two dimensions of the ANPS: ANGER and CARING. Although these two ANPS scales were not significantly related, they both correlated in opposite directions (positively with CARING and negatively with ANGER) with the Agreebleness construct [2], [10], [11]. Consequently, Agreebleness appears to be a personality trait associated with at least two core emotional systems.

Since the initial validation study by Davis et al. [2], several studies have provided additional evidence to consider that the ANPS scores can be validly interpreted for the characterization of core emotional endophenotypes – with validity evidence derived from psychometric, neural, clinical and genetic approaches [10]–[19].

### Rationale for a Short Version of the ANPS and Hypotheses

The purpose of the present study was to propose a short version of the six ANPS scales: SEEKING, CARING, PLAYFULNESS, ANGER, FEAR, and SADNESS. A SPIRITUALITY scale was introduced in the ANPS ‘*for a hypothesized higher-order affective human attribute*’ [2]. However, as it was not based on Panksepp and coll. neuro-ethological model, we excluded this scale from the present analyses. In the long version, each scale comprised 14 items, resulting in an 84-item questionnaire. Davis et al. [2] stated that their intention was ‘*eventually to reduce the scale to ten items per category’* (p. 60). However, ten items per category (i.e. 60 in total) could still prevent use of the instrument in large samples, in particular in longitudinal studies where subjects are followed at each assessment on a whole range of characteristics, and among clinical patients for whom answering a long questionnaire could be too demanding. Furthermore, the list of core emotions proposed by Panksepp [4] is open and could be completed or refined with additional progress in the neurosciences (for instance this author has already proposed LUST, i.e. an emotional system linked to erotic desire). Hence, a short version of the ANPS could easily be completed with other personality dimensions and still remain acceptable in length.

Here, we aimed to reduce the instrument to six items per scale, for a total of 36 items. The choice of six items was a compromise between pragmatic considerations (e.g., time and cost of completion on large samples) and the need to obtain acceptable psychometric properties, in particular internal consistency coefficients which decrease with the number of items [20].

We expected that the properties of the short and the long version would be similar, in particular regarding internal consistency, intercorrelations, factorial structure and gender differences. Regarding gender differences, CARING and SADNESS scores among women were found to be higher than scores among men in four previous studies [2], [10], [11], [13]. Three studies found that women scored higher on FEAR [10], [11], [13] and two that men scored higher on PLAYFULNESS [11], [13]. We expected to replicate these findings.

In previous studies, the Cronbach’s alpha for each of the instrument scales appeared satisfactory, ranging from.63 to.89 [2], [10], [11], [13] **.** We expected that the removal of items in the short version would not lower these coefficients too much. We also expected to replicate the intercorrelations patterns reported in previous studies, in particular the positive correlations between the three positive scores (PLAYFULNESS, SEEKING, CARING) and linking the three negative scores (FEAR, ANGER, SADNESS). Furthermore, all studies found a positive correlation linking CARING, FEAR and SADNESS scores.

Three previous studies [10], [11], [13] conducted a Confirmatory Factor Analysis on the instrument. The analyses revealed that the goodness of fit indices were satisfactory but also indicated that the factorial structure of the instrument could be improved, suggesting in particular that some scales were not completely unidimensional [13]. We expected that, in the short version, the unidimensionality of the different scales would be verified. Another concern regarding the factorial structure of the long version was the overlap between the FEAR and SADNESS scales [2], [10], [11], [13], [16]. Hence, our objective was to minimize this overlap and, consequently, we expected lower correlation between FEAR and SADNESS.

Finally, we expected that, for each scale, the scores of the short and long version would be highly correlated.

## Materials and Methods

### Participants

Two samples were used in this study. The first included 830 French participants. The participants were studying or working in various fields (social workers, psychology, art, biology and biotechnologies, computer science, and general engineering). One thousand questionnaires were distributed and completed during classes (by the students and the teachers). Eight-hundred and sixty-nine questionnaires were returned, of which 32 had a partially uncompleted consent form (either identity or signature was missing, although the questionnaires were completed). For the 837 remaining participants, seven had not answered all the ANPS items, leading to a final sample of 830 healthy young adults (54.8% women; mean age of the men = 20.69 years, SD = 2.32; mean age of the women = 20.56 years, SD = 1.99), with an intermediate to high level of education (31.2% graduated from high school, 29.6% with less than 2 years of college, 39.2% with more than 2 years of college). Only two participants did not complete high school: they were porters who were present when the questionnaires were distributed and who were willing to participate. Among these participants, a subset of 430 (52.3% women) subjects was randomly selected (using the sample() command from R software [21]) and used for item selection; the remaining participants constituted a second subset (N = 400; 57.5% women) and served for an analysis of the psychometric properties of the Affective Neuroscience Personality Scales short version (ANPS-S, see data analysis). A sample size greater than 300 is considered adequate for the internal validation of psychiatric scales [22].

The second sample included 431 Canadian participants with a 5-year-old child who were part of a longitudinal study on child development (60.6% women; mean age of the men = 38.14 years, SD = 6.39; mean age of the women = 35.00 years, SD = 4.94). Among the mothers, only 0.9% did not have a high-school diploma, 22.0% had a high-school diploma, 20.6% a post-secondary diploma other than university diploma and 56.5% a universitary diploma. These percentages were respectively, 3.5%, 25.7%, 16.7% and 54.2% for the fathers.

### Language

We used the French version of the scales (ANPS 2.4) as validated by Pahlavan et al. [11]. These authors translated the ANPS into French and back into English (the original language of the instrument) to ensure an adequate translation. In the present study, we also used this French instrument for participants in Quebec. As some expressions differ in Canadian French and French, the third author, who is a native French Canadian, reviewed the items and concluded that they could be understood without modification.

### Item Selection and Data Analyses

As mentioned in the introduction, the overlap between the SADNESS and FEAR scales was a concern [13]. We decided to select the items for these two scales on the basis of a factorial analysis using maximum likelihood. We extracted two factors and performed a subsequent oblique (oblimin) rotation – see Clark and Watson [23] for a similar procedure. Furthermore, four items of the CARING scale, all about the respondent's feelings towards animals, seemed to introduce heterogeneity in the scale and were excluded from the selection.

To select the items for the short version we first computed a series of values for each item:

- A loading for each item from a one factor analysis of each scale;
- Item-total correlation: the coefficient of correlation between each item of a given scale and the total score of this scale;
- A measurement of any loss or gain in the Cronbach alpha associated with the removal of an item: Cronbach alpha for the remaining items minus Cronbach alpha for the whole scale.
- Unbalanced items: highly unbalanced items (e.g. almost all subjects choosing the “strongly agree” response option for an item) are undesirable [23]. The percentage of subscribers to each response depends on the number of response options available (here, with four response options, a uniform distribution would imply 25% for each response option). Hence, items with more than 75% of the participants subscribing to one of the four response options were excluded.

The decision regarding the 6 items retained for each shortened scale was based on consideration of all the aforementioned values. In addition, a close comparison of the content of the selected items and the items available in the full version was conducted in order to ascertain that the content explored in the full version was considered in the short version. When the aforementioned selection methods did not fully converge, the final choice was based on the content argument. For instance, items concerning children were considered more central, theoretically, to the CARING scale than an item concerning homeless people. Indeed, given the animal model underlying the scale, caring would be directed more towards related children than strangers [4].

On the two confirmation data sets (i.e. French subsample 2, N_2_ =  400; Canadian sample, N_3_ =  431), the following analyses were conducted in order to confirm the properties of the short version of the instrument:

- Skewness, kurtosis, ceiling and floor effects. Regarding, ceiling and floor effects, a maximum of 15% of the participants should choose the floor and ceiling option responses in the final scale [24].
- Internal consistency coefficients. Clark & Watson [23] emphasized that the average interitem correlation is a straightforward measure of internal consistency. Average interitem correlation should fall in the range of 15–50. According to the same authors, the Cronbach’s alpha is an ambiguous measure of internal consistency because it also depends on the number of items; however, it conveys important information as to the proportion of error variance contained in the scale. We present the two coefficients.
- Intercorrelations (Pearson coefficients) linking the ANPS-S scores;
- Unidimensionality the different scales. To assess the unidimensionality of the scales, we used parallel analysis, i.e. a graphic representation of the eigenvalues with simulated data sets having the same number of variables and subjects [25].
- Confirmatory Factor Analysis (CFA) of the whole instrument with 6 correlated factors corresponding to the 6 ANPS-S scales. Regarding the interpretation of the CFA fit indices, we chose to follow the recommendations of Kline [26] and Bentler [27] and used the three indices they proposed and their commonly reported cutoffs: SRMR (Standardized Root Mean Square Residual) which should remain under.10, RMSEA (Root Mean Square Error of Approximation) under.05 and the CFI (Comparative Fit Index) above.90. We also specified the pathways introduced into the model after an analysis of the residuals [27].
- Gender differences. We expected to replicate a significant effect at least for the CARING, SADNESS and FEAR scales which were the most consistent and/or strongest previous findings.

## Results

### Final Item Selection and Short Scale Content

For the FEAR and SADNESS items, the two-factor analysis with oblique rotation revealed that the six items with the highest loading on the first component were SADNESS items, and the six items with the highest loading on the second component were FEAR items. These six SADNESS and FEAR items were retained.

Regarding the ANGER scale, the main selection procedures (i.e. one-factor analysis, item-total correlation, loss in Cronbach alpha) agreed for six items and none had high ceiling or floor effects. These 6 items were therefore retained.

For CARING, the main procedures agreed for 5 items. For the sixth item, there were two potentially eligible items: “*I do not especially like being around children*” and “*I am the kind of person that likes to touch and hug people*”. This second item had crossloadings on other components (e.g., negative crossloading with SADNESS); this was not the case for the item “*I do not especially like being around children*” which was therefore retained.

Regarding the SEEKING scale, 5 items were designated by all three main selection procedures. For the sixth item, the choice involved three potentially eligible items : a) “*I do not get much pleasure out of looking forward to special events*”; b) “*I am usually not interested in solving problems and puzzles just for the sake of solving them*”; c) “*I rarely feel the need just to get out and explore things*”. Given the neuro-ethological background of the scale, we chose to keep item (c) which relates to exploration. In addition, it does not include the word “problem” which was found to be an issue in a previous study because of residual correlations of items containing this word with the FEAR and SADNESS scales (Pingault et al., 2011). Consequently, the short version of the SEEKING scale contains core dimensions of the original concept which are curiosity, exploration and novelty.

The main procedures agreed on 5 items for the PLAYFULNESS scale. However, one of them had a high ceiling effect (over 75%) and was therefore removed. To replace this fifth item, two items were potentially eligible by the main procedures: a) “*I generally do not like vigorous games which require physical contact*” and b) “*I do not tend to see the humor in things many people consider funny*”. Item (a) seemed potentially confusing because of the word ‘violent’ is used in the French translation, and quite unnecessary, as the physical dimension of play was already present in another item selected for the short version. Instead, for the fifth item, we retained the item relating to sense of humour (b). For the sixth item of the short PLAYFULNESS scale, we selected another item which showed good scores on each procedure, and its content was considered a priori essential to this scale as theorized by Panksepp (2006) (i.e., being joyful) : “*I am a person who is easily amused and laughs a lot*”.

### Psychometric Properties of the ANPS Short-form

The analyses were conducted on the two confirmation samples (N_2_ and N_3_). Table 1 provides norms for women and men in these two samples. Skewness and kurtosis values were low (skewness ranging from −.53 to.56 and kurtosis ranging from −.47 to.43; Table 2). Average interitem correlation ranged from.22 to.39 in both samples (Table 2). Cronbach’s alphas ranged from.60 to.79 in both samples (Table 2). No ceiling or floor effects were detected. The maximum percentage in a floor or ceiling response option (i.e., 0 or 18 as the scales contain 6 items, each one coded from 0 to 3) was 5.5% in the French confirmation sample (score 18 for the PLAYFULNESS scale) and 5.3% in the Canadian sample (score 18 for the SEEKING scale), which is well below the acceptable percentage [24].

**Table 1:** Descriptive Scores of the Short Version of the Affective Neuroscience Personality Scales by Sex.

	Men			Women			Sex differences
	Min-Max	Mean (SD)	Median (IQR)	Min-Max	Mean (SD)	Median (IQR)	Cohen’s *d*
*French sample (N_2_)*							
PLAYFULNESS	(0–18)	13.26 (2.88)	14.00 (12–15)	(4–18)	12.77 (2.99)	13.00 (11–15)	0.17
SEEKING	(5–18)	13.18 (2.83)	13 (11–15)	(6–18)	13.08 (2.60)	13 (11–15)	.04
CARING	(2–17)	10.05 (3.15)	10 (8–12)	(0–18)	12.09 (3.26)	13 (10–14)	−.64***
FEAR	(0 −17)	7.85 (3.47)	8 (5–10)	(2–18)	10.24 (3.43)	11.00 (8–12)	−.70***
ANGER	(0–17)	7.91 (3.76)	8 (5–10)	(0–18)	8.5 (4.10)	8.5 (6–11)	−.15
SADNESS	(0–17)	7.22 (3.73)	7 (4.25–10)	(0–17)	8.26 (3.48)	8 (6–11)	−.29**
*Canadian sample* *(N_3_)*							
PLAYFULNESS	(6–18)	12.74 (2.63)	13 (11–15)	(3–18)	12.22 (2.77)	12 (10–14)	.20*
SEEKING	(4–18)	13.01 (2.98)	13 (11–15)	(1–18)	12.68 (2.93)	13 (11–15)	.11
CARING	(4–17)	11.67 (2.64)	12 (10–14)	(5–18)	12.77 (2.57)	13 (11–15)	−.42***
FEAR	(0–14)	6.34 (3.06)	7 (4–8)	(0–18)	8.51 (3.29)	8 (6–11)	−.68***
ANGER	(0–17)	6.84 (3.49)	7 (4–9)	(0–17)	7.51 (3.28)	7 (5–10)	−.20*
SADNESS	(0–16)	5.25 (3.02)	5 (3–7)	(0–16)	6.7 (3.14)	6 (5–8)	−.47***

*Note.* Analyses were conducted in a French sample (N_2_ = 400; 170 men) and a Canadian sample (N_3_ = 431; 180 men). For each scale and each sex the table presents the minimum and maximum, the mean and standard deviation (SD), the median and the interquartile range (IQR). The last column indicates the size effects (Cohen’s *d*) for the differences between men and women, as well as the significance of the difference based on a t-test.

*** p<.001;

** p<.01;

* p<.05.

**Table 2:** Average inter-item correlation, Cronbach’s alpha, Skewness and Kurtosis of the Affective Neuroscience Personality Scales Short Version.

	Interitem correlation	Alpha	Skewness	Kurtosis
*Fench sample (N_2_)*				
PLAYFULNESS	.27	.68	−0.53	0.43
SEEKING	.25	.61	−0.32	−0.41
CARING	.28	.70	−0.37	−0.19
FEAR	37	.77	0.05	−0.47
ANGER	.39	.79	0.21	−0.47
SADNESS	.34	.75	0.22	−0.33
*Canadian sample (N_3_)*				
PLAYFULNESS	.23	.60	−0.29	−0.24
SEEKING	.30	.70	−0.42	0.23
CARING	.22	.60	−0.37	−0.06
FEAR	.36	.77	0.12	−0.12
ANGER	.36	.77	0.18	0.01
SADNESS	.30	.71	0.56	0.25

All three negative scales (FEAR, SADNESS and ANGER) had Cronbach’s alphas above.70 in both samples. However the three positive scales failed to reach.70 in one or the other sample (Table 2). The CARING coefficient was.70 in the French confirmation sample but.60 in the Canadian sample whilst the reverse was true for the SEEKING score (.61 in N_2_;.70 in N_3_). The PLAYFULNESS coefficient was.68 in the French confirmation sample and.60 in the Canadian sample.

Table 3 presents the intercorrelations between the scale scores in both samples (N_2_ and N_3_). The main results are that, in both samples, the three positive scores (PLAYFULNESS, SEEKING and CARING) were positively intercorrelated and so were the three negative scores (FEAR, ANGER, SADNESS). Furthermore, the CARING score was positively correlated to the FEAR score in both samples, as well as to the SADNESS score in the Canadian sample.

**Table 3:** Intercorrelations Between the Affective Neuroscience Personality Scales Short Version Scores.

	PLAYFULNESS	SEEKING	CARING	FEAR	ANGER
*French* *sample (N_2_)*					
SEEKING	.35***				
CARING	.28***	.15**			
FEAR	−.20***	−.11*	.13**		
ANGER	−.07	.06	−.07	.24***	
SADNESS	−.28***	−.10*	.08	.47***	.24***
*Canadian* *sample (N_3_)*					
SEEKING	.31***				
CARING	.31***	.18***			
FEAR	−.22***	−.15**	.20***		
ANGER	−.12*	−.02	−.04	.34***	
SADNESS	−.17***	−.03	.17***	.52***	.32***

*** p<.001

** p<.01

* p<.05.

**Figure 1:**
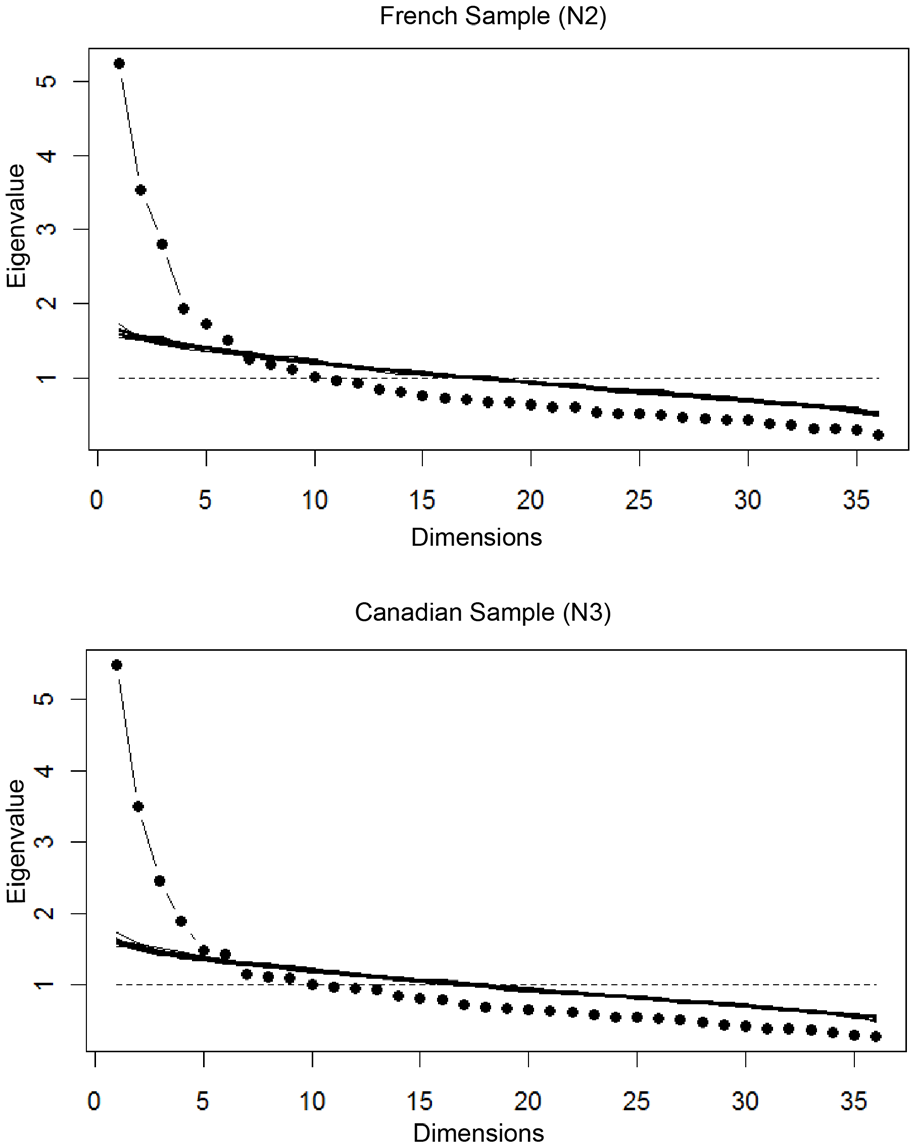
Title: Number of dimensions in the ANPS-S in the French Sample (N2) & the Canadian Sample (N3). Legend: Representation of eigenvalues with simulated data sets: six dimensions emerge (above the simulation lines) for the Affective Neuroscience Personality Scales Short Version.

Parallel analysis showed that all scales in the short version were unidimensional. The only exception was for the PLAYFULNESS scale in the Canadian sample, with one additional eigenvalue point slightly above the simulation line, but it was unidimensional in the French sample**.** The same analysis was conducted again with the 36 items of the whole instrument, showing that 6 dimensions emerged in both samples (see Figure 1). In addition, a graphic confirmatory analysis (available on request from the authors) showed that the median of the correlations between the items of a given scale was always greater than the 5 medians of the correlations linking items in that scale to items in each of the other scales. This result holds true for each of the 6 scales.

Regarding the CFA, the fit indices were identical in both samples: SRMR.06, RMSEA.04 and CFI.90. The residuals entered into the model were only related to residual covariance between items in the same scale (for instance two items in SEEKING). Therefore, no residuals introduced into the final model jeopardized the theoretical structure of the instrument.

The correlations between the scores of the short and the long version for each scale were high in both samples, with values falling between.79 and.92 (Table 4). Finally, in both confirmation samples, women scored significantly higher for CARING, FEAR and SADNESS (Table 1) with small or medium effect sizes. In the Canadian sample, men scored higher on PLAYFULNESS and women higher on ANGER, but with small effect-sizes. A list of the items selected in the ANPS Short Version for each scale is provided in the Supporting information S1.

**Table 4:** Correlations Between The Long and Short Versions of the Affective Neuroscience Personality Scale Scores.

	French subsample *(N_2_)*	Canadian sample *(N_3_)*
PLAYFULNESS	.92	.91
SEEKING	.81	.84
CARING	.79	.79
FEAR	.87	.88
ANGER	.90	.88
SADNESS	.87	.87

## Discussion

The ANPS was designed as a tool to assess endophenotypes related to activity in the subcortical brain emotional systems that help to generate key components of affective experience in all mammalian species [2], [4], [8], [11]. Previous studies provided arguments for considering the ANPS as a promising tool in a multilevel approach integrating genes, brain and psychiatric distress [10]–[19]. The aim of this article was to propose a short version of the Affective Neuroscience Personality Scales [2]. We obtained an instrument comprising 36 items, 6 for each of the following scales: FEAR, SADNESS, ANGER, SEEKING, CARING, and PLAYFULNESS.

We compared the properties of the ANPS-S to those of the long instrument and found that they were very similar. Skewness and kurtosis values for all the scores of the short version were close to zero in both samples, replicating previous findings with the long version [13]. These values are a sign of a distribution close to normality and mean that the scores can be used without transformation (e.g. logarithmic transformation) in a wide range of analyses. For instance, in a Structural Equation Modeling analysis with Maximum Likelihood estimation, values up to 2 for skewness and 7 for kurtosis are still acceptable [28].

Average inter-item correlation ranged from.22 to.39. Clark & Watson [23] suggested that values between.15 and.50 were indicative of acceptable internal consistency. They emphasized that narrow constructs (e.g. fear of intimacy) should have higher average interitem correlation (e.g. 40–.50). In broad constructs (e.g., positive affects), average inter-item correlation should remain fairly low (e.g. 15–.20) to adequately capture the full construct. The scales in the ANPS are intermediate, ie. neither very wide nor narrow. As such, the values ranging from.22 to.39 found in the present study seem acceptable. Previous studies of the ANPS did not present average inter-item correlations, so that we encourage other researchers to provide them in future research or to re-examine existing data sets.

As emphasized by Clark & Watson [23], Cronbach’s alpha conveys information on the proportion of error variance contained in the scale (rather than on internal consistency). Cronbach’s alphas ranged from.60 to.79 in both samples, which represented a surprisingly moderate loss compared to a range from.65 to.86 in the orginal long version, given that Cronbach’s alpha decreases with the number of items [20]. Coefficients were above.70 for the short negative scales in the two confirmation samples. Conversely, for the three positive scales (SEEKING, CARING and PLAYFULNESS) the coefficient was below.70 in at least one of the two confirmation samples. Thus, future studies using the ANPS-S should help to determine whether, depending on sample fluctuations, Cronbach’s alphas are under.70 for these three scales. At the same time, Lance, Butts, & Michels [29] noted that Nunnally [30], the source of this commonly reported cutoff, did not make a standard of the.70 cutoff, but proposed that the value should depend on how a measure is being used. As emphasized by Schmitt [20], in case of low alpha coefficients, the concern : *'is that the true correlations involving a predictor and an undereliable outcome variable will be serioulsy attenuated (i.e. underestimated) because of inadequate criterion reliability rather than any lack of real or true relationship […] With reliability equal to.70, validity has an upper limit of.84 (i.e., the square root of.70) as opposed to 1.00. Even with reliability as low as.49, the upper limit of validity is.70′*. Therefore, with an alpha of.60, as in the present case, the upper limit is.77 compared with an upper limit of.84 with an alpha of.70, which represents a moderate loss.

The rationale for proposing a short version was in particular to provide an instrument for large samples. On such samples, Cronbach’s alpha should not be a detterant as the power to detect existing correlations with other variables is high. On this issue, Schmitt concludes that : ‘*When a measure has other desirable properties, such as meaningfull content coverage of some domain and reasonable unidimensionality, this low reliability may not be a major impediment to its use*’ [20]. The ANPS-S fulfils these requirements (see below).

Intercorrelations between the ANPS-S scores were highly similar to those observed with the long version. The positive correlations between the three positive scales scores on the one hand, and the three negative scales scores on the other hand, replicated the previous findings with the long version [2], [10], [11], [13]. We also replicated the positive correlation between the CARING score with both the FEAR and SADNESS scores (except in the French sample for CARING and SADNESS). As in previous studies [2], [11], [13], but not in [10], we found small negative correlations of the PLAYFULNESS score with both the FEAR and SADNESS scores in the French and Canadian samples. We also found some differences with results reported for the long version. First, and in line with the intention to reduce the overlap between the FEAR and SADNESS scales, the correlation between these two scale scores was reduced: around.50 with the short version against.57 to.73 with the long version in the different studies [2], [10], [11], [13], [16]. The other difference was the negative correlations observed between the SEEKING score and both the FEAR and SADNESS scores. However, these correlations were low (≤.15) and not consistent in the two samples.

The factorial structure of the ANPS-S was good. Indeed, all scales were clearly unidimensional, which enhances their interpretability (on the importance of unidimensionality, see [20], [23]). The unidimensionality of the six scales has another advantage: it enables correction for attenuation resulting from Cronbach’s alpha (to provide accurate estimates of the relationship between constructs) [20]. Furthermore, regarding the overall factorial structure, the instrument clearly possessed 6 dimensions and all the goodness-of-fit indices of the two CFA were in agreement with the commonly reported cutoffs.These results are of importance, as the indices are better than in similar analyses conducted on the long version in previous studies [11], [13].

Additionally, a content analysis demonstrated that the core dimensions of the long version of the scales were retained in the short version. With respect to the CARING scale, items asking about the respondent's feelings towards animals, which introduced heterogeneity into this scale, were discarded. From an ethological point of view, intra and inter-specific interactions, even if they share morphological characteristics, do not necessarily obey to the same function or derive from the same neurological basis, as illustrated, for instance, by the difference between predation and agression [31]. Thus, feelings towards animals may be more a mere consequence of morphological similarities between animal offsprings and human babies than a pertinent way to assess proneness to CARING in an individual. For the FEAR scale, items representing both fear and anxiety were selected. Items assessing the difficulty to make decisions or anxiety about past decisions were not represented, but they can be considered of lesser theoretical importance. In forthcoming studies, the FEAR scale could possibly be splitbetween fear and anxiety components if the litterature provides additional evidence that they are two separate dimensions. Indeed, some authors argue that, although fear and anxiety behaviours have usually not been distinguished and both share functions dealing with facing danger, they may have different – though partially overlapping – neurological underpinnings and respond to different drugs [32]–[34]. This is an additional strength of the theoretical approach used for the ANPS: two behaviors that manifest themselves in similar contexts and are usually not distinguished could be distinguished on the basis of their neurological underpinnings and response to drugs. As a consequence, personality assessment could become less dependent on lexical, psychometric and/or phenotypical definitions.

In the short version of the SADNESS scale, sadness itself and the loss of “loved ones”, which are the two core elements of the scale, were represented. None of the items relating to mere friends were represented, which is interesting, as the SADNESS scale has been theorezed mainly from animal literature on the loss of attachment figures, which are necessarily close relatives [8]. The content of the three other short scales – PLAYFULNESS, SEEKING, ANGER – also adequately reflected the content of the long version.

In addition, all correlations between the short and the long scale scores on both samples were high, showing that the ANPS-S can be interpreted as representing the original content of the scales. The only correlation under.80 was for the CARING scale (.79 in both confirmation samples), which may be due to the removal of the items relating to animals which initially introduced heterogeneity in the scale. However, the items representing the core theoretical bases of this scale were retained.

Finally, gender differences were very similar to those found previously with the long version of the instrument [2], [10], [11], [13]. Indeed, differences of small to medium effect sizes were found in both samples for the CARING, FEAR and SADNESS scales (women scoring higher). This is in line with the reported evidence that women display greater propensity for nurturing and empathizing than men [35], [36] and is consistent with clinical studies showing that depression and anxiety disorders are more common in women than men [37]–[39]. In addition, men scored higher on PLAYFULNESS, but the effect was small and significant only in the Canadian sample.

Overall, the short version of the ANPS demonstrated its consistency with the long version in two different samples: content and psychometric analyses showed that each scale of the short version can be considered as a proper assessment of the dimension it is supposed to represent. Furthermore, the factorial structure of the ANPS-S fitted the theoretical structure of the instrument better than the long version. The main weakness of the ANPS-S was the possibility of low Cronbach’s alphas for the three positive scales, depending on the sample. However, we proposed a short version to make the instrument available in particular for studies with large samples in order to reduce the time and cost of completion. On these samples, as discussed earlier, this issue should not be a deterrant for the use of the instrument. We believe that the ANPS-S represents an interesting alternative to the full version when the length of the full version would prevent its use.

### Limitations and Strengths

Although convergent and discriminant evidence has been collected for the ANPS – to which the ANPS-S has been shown to be closely related – further evidence of this type should be collected for the short form [40]. Furthermore, the correlations between the long and short scales scores on the basis of one test administration may be overestimated [40]. Therefore, both forms should be administered separately to the same participants before calculating more reliable estimates of the correlations between short and long scale scores. The main weakness of the ANPS-S is the Cronbach alphas for the positive scales, which were adequate or low depending on the sample. We encourage additional studies in other French populations or sub-populations to address this question. Furthermore, we constructed the ANPS-S with French speaking participants. Given that the long version was first developed in the United States and has been successfully validated in several languages, we expect that the ANPS-S will behave in a similar fashion in other languages. However, this will require a formal confirmation and we encourage other researchers to test the properties of the ANPS-S in other languages.

We examined the properties of the ANPS-S in two samples of size (N>300) which is considered adequate for the internal validation of psychiatric scales [22]. Furthermore, these samples had different characteristics in terms of age and level of education, and were from two different countries which, even if they share the same language, are culturally diverse. Despite these differences, the marked similarity of the results strengthens our confidence in the properties of this short version of the ANPS. The theoretical framework behind the ANPS as well as its international collaborative development has warranted its use in a wide range of studies, namely epidemiological, clinical, imaging and genetic studies. We believe that the short version of the ANPS, which is also free access, will be useful in a wide range of research designs; the ANPS-S will be of particular interest for large cross-sectional or longitudinal studies or any other research design where questionnaire length is an issue, as well as for clinical patients who might not answer long questionnaires. As such, our research provides a valuable addition to the existing measures of personality and emotion and we encourage other researchers to pursue the validation and use of the ANPS-S.

## Supporting Information

Supporting Information S1 **Items selected in the ANPS Short Version for each scale.**
(DOCX)
