# Supplementary material for: A New Approach of Personality and Psychiatric Disorders: A Short Version of the Affective Neuroscience Personality Scales
Source: PLoS One. 2012 Jul 26;7(7):e41489. doi: 10.1371/journal.pone.0041489 (PMC3406066; doi:10.1371/journal.pone.0041489)
Supplement: Supporting Information S1 — Items selected in the ANPS Short Version for each scale. (DOCX) [file pone.0041489.s001.docx]

## Supporting information file S1

Items selected in the ANPS Short Version for each scale:

SEEKING:

I really enjoy looking forward to new experiences.

I am usually not highly curious.

My curiosity often drives me to do things.

I rarely feel the need just to get out and explore things.

Whenever I am in a new place, I like to explore the area and get a better feel for my surroundings.

I am not an extremely inquisitive person.

CARING:

I often feel a strong need to take care of others.

I like taking care of children.

Caring for a sick person would be a burden for me.

I do not especially like being around children.

I am a person who strongly feels the pain of other people.

I am not particularly affectionate.

PLAYFULNESS:

I am a person who is easily amused and laughs a lot.

I do not particularly enjoy kidding around and exchanging "wisecracks."

I am very playful.

I do not tend to see the humor in things many people consider funny.

I like all kinds of games including those with physical contact.

Playing games with other people is not especially enjoyable for me.

ANGER:

When I am frustrated, I usually get angry.

My friends would probably describe me as hotheaded.

When I am frustrated, I rarely become angry.

People who know me well would say I almost never become angry.

I hardly ever become so angry at someone that I feel like yelling at them.

When people irritate me, I rarely feel the urge to say nasty things to them.

FEAR:

People who know me well would say I am an anxious person.

I am not frequently jittery and nervous.

I would not describe myself as a worrier.

I have very few fears in my life.

My friends would say that it takes a lot to frighten me.

There are very few things that make me anxious.

SADNESS:

I often feel sad.

I often have the feeling that I am going to cry.

I rarely become sad.

I often feel lonely.

I often think about people I have loved who are no longer with me.

I tend to think about losing loved ones often.
